# Detection of Amitraz Resistance in *Rhipicephalus (Boophilus) microplus* from SBS Nagar, Punjab, India

**DOI:** 10.1155/2014/594398

**Published:** 2014-02-11

**Authors:** N. K. Singh, Abhijit Nandi, Jyoti, S. S. Rath

**Affiliations:** Department of Veterinary Parasitology, College of Veterinary Science, Guru Angad Dev Veterinary and Animal Sciences University, Ludhiana 141004, India

## Abstract

The resistance status of *Rhipicephalus (Boophilus) microplus* collected from SBS Nagar, Punjab, was evaluated against amitraz by Adult Immersion Test (AIT). The regression graph of probit mortality of ticks plotted against log values of progressively increasing concentrations of amitraz revealed the slope of mortality (95% CI) as 2.36 ± 0.61 (0.38 to 4.33). The LC_50_ (95% CI) and LC_95_ (95% CI) values were recorded as 332.52 ppm (305.06–362.44) and 1646.93 ppm (1383.97–1959.84), respectively, and the resistance factor (RF) was 13.17 which indicated level II resistance status. The dose response curves for egg mass weight, reproductive index, and percentage inhibition of oviposition were also validated by AIT and the slopes (95% CI) were −7.17 ± 2.41 (−14.85 to 0.50), −0.009 ± 0.02 (−0.16 to −0.031), and 19.99 ± 4.77 (4.81 to 35.17), respectively. The current study reports the development of resistance in *R. (B.) microplus* to amitraz from Punjab state and the data generated would be useful in formulation of effective control strategies against ticks of this region.

## 1. Introduction

*Rhipicephalus (Boophilus) microplus* is a widely prevalent tick infesting dairy animals in tropical and subtropical regions of the world, causing major economic losses to cattle producers directly through feeding on parasitized cattle and indirectly by transmitting several disease-causing pathogens to the host (*Babesia bovis*, *Babesia bigemina* and *Anaplasma marginale*) [1]. One of the most employed strategies used to control infestation of *R. (B.) microplus *is the application of acaricides on the body of the host. However, the indiscriminate and incessant use with improper concentrations has probably contributed to the development of resistance to most of the acaricides in several countries [2].

The formamidine (amitraz) was initially introduced to control organophosphate (OP) resistant ticks at the same time when the synthetic pyrethroids (SP) were introduced, but its use was initially limited due to higher cost. However, the development of SP resistance in ticks has led to an increased dependency of farmers on amitraz and hence its usage has increased many folds in recent past. In India, the first case of amitraz resistance was detected in *R. (B.) microplus* ticks from Banaskantha district, Gujarat [3]. Further, the development of tick resistance against the OP compounds [4] and SPs [5] in Punjab state had caused the farmers to shift towards indiscriminate use of amitraz for tick control, thus leading to emergence of resistance against it. However, as reports of development of amitraz resistance in *R. (B.) microplus* are unavailable from this part of the country, the current study was undertaken to detect amitraz resistance in *R. (B.) microplus*.

## 2. Materials and Methods

### 2.1. Sample Collection

Live engorged adult female *R. (B.) microplus* ticks were collected from sheds of dairy animals comprising cross bred cattle as well as buffaloes from district SBS Nagar, Punjab. Also data related to frequency, type and mode of acaricide treatment adopted by the owners and their experiences about the commonly used acaricides efficacy were recorded. The ticks were collected in vials, closed with muslin cloth to allow air and moisture exchange, brought to the Entomology Laboratory, Department of Veterinary Parasitology, GADVASU, Ludhiana, cleaned, labelled and kept at 28 ± 1°C and 85 ± 5% relative humidity.

### 2.2. Acaricide

Technical grade amitraz 100% pure (AccuStandard Inc. U.S.A) was used to prepare the stock solution in methanol. For the experimental bioassay, various concentrations of amitraz were prepared in distilled water from the stock solution and tested against the field isolate.

### 2.3. Adult Immersion Test (AIT)

Adult immersion test was conducted according to the method of Sharma et al. [6] with minor modifications. Briefly, the preweighed engorged females of *R. (B.) microplus* were immersed in various concentrations of amitraz (62.5, 125, 250, 500 and 1000 ppm) for 2 min and then dried on filter paper before transferring into the Petri dishes. After 24 h, ticks were transferred to the glass tubes covered with muslin cloth and were kept in desiccators kept in BOD incubator maintained at 28 ± 1°C and 85 ± 5% RH. The ticks which did not oviposit even after 14 days after treatment were considered as dead. The control group was treated in similar manner in distilled water. Each concentration was replicated twice and ten adults were used per replication and the following parameters were compared:

- mortality: recorded up to 14 days after treatment (dpt),
- the egg masses laid by the live ticks,
- reproductive Index (RI) = egg mass weight (EW)/engorged female weight (EFW),
- percentage inhibition of oviposition (IO%) = ((RI control − RI treated)/RI control × 100).

Dose response data were analyzed by probit method [7] using Graph Pad Prism 4 software. The lethal concentration for 50% (LC_50_) and LC_95_ values of amitraz was determined by applying regression equation analysis to the probit transformed data of mortality.

### 2.4. Resistance Diagnosis

Resistance factor (RF) was worked out as per the method of Singh et al. [3]. On the basis of RF, the resistance status was determined as susceptible (RF < 1.4), level I resistant (RF = 1.5–5.0), level II resistant (RF = 5.1–25.0), level III resistant (RF = 25.1–40), and level IV resistance (RF > 40.1) as per Sharma et al. [6].

## 3. Results and Discussion

The response of *R. (B.) microplus* collected from SBS Nagar, Punjab, with various concentration of amitraz in terms of mortality, egg mass weight, reproductive index (RI) and percentage inhibition of oviposition (IO%) is presented in Table 1. The mortality of ticks showed an increasing trend along with the increasing concentrations of amitraz and maximum mortality of 80.0% was recorded at the highest concentration of 1000 ppm. It was further observed that the concentration at which amitraz is being widely used (250 ppm) in field conditions could only achieve 40.0% mortality and even much higher concentration of 1000 ppm failed to produce 100% mortality, thus indicating development of resistance against amitraz in these ticks.

**Figure 1:**
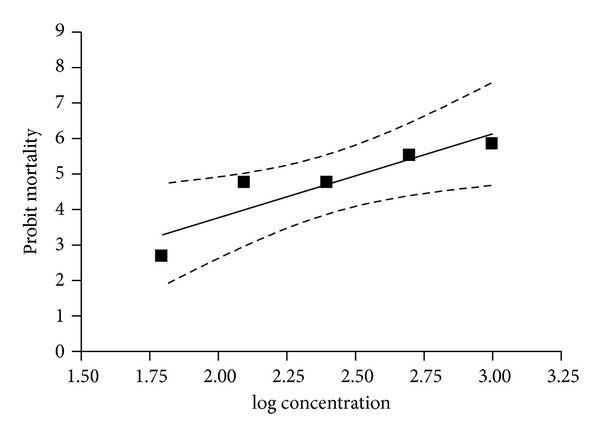
Dose mortality curve of *R. (B.) microplus *against amitraz.

The regression graph of probit mortality of ticks plotted against log values of progressively increasing concentrations of amitraz is shown in Figure 1. The dotted lines in the regression curve represented the 95% confidence limits. The slope of mortality (95% confidence intervals) was 2.36 ± 0.61 (0.38 to 4.33), whereas the value of goodness of fit (*R* ^2^) was 0.8288. From the regression equation the values of LC_50_ (95% CI) and LC_95_ (95% CI) were recorded as 332.52 ppm (305.06–362.44) and 1646.93 ppm (1383.97–1959.84), respectively, and the RF was 13.17 which indicated level II resistance status.

The dose response curves of *R. (B.) microplus *against log values of progressively increasing concentrations of amitraz were plotted for egg mass weight, reproductive index and IO% by the data generated by AIT. The average egg mass weight of ticks treated with different concentrations of amitraz decreased with increasing concentrations of drug but the variation was statistically non-significant (*P* = 0.0589). The slope (95% CI) of egg mass weight was −7.17 ± 2.41 (−14.85 to 0.50) and was negative because, with the increasing concentrations of acaricide, ticks died. The RI of ticks treated with different concentrations of amitraz decreased with increasing concentrations of drug and the slope (95% CI) was −0.099 ± 0.02 (−0.16 to −0.031) indicating that, although the increase in concentration of the acaricide may have not caused 100% mortality, the survived ticks showed decrease in their efficiency to convert the live weight into egg mass. Further, there was an increase in the IO% in ticks with increase in drug concentration and thus a positive slope (95% CI) of 19.99 ± 4.77 (4.81 to 35.17) was recorded.

Among the various acaricides used in India for the control of ticks in livestock, resistance has been reported against most of the acaricides in *R. (B.) microplus* [4–6, 8–10]. Reports of resistance against amitraz are available against *R.* (*B.) microplus* ticks from various parts of world [11, 12] but only single report of amitraz resistance in *R. (B.) microplus* is currently available from Gujarat, India [3]. Further, resistance against amitraz has not been reported from Punjab state, probably because the use of amitraz for tick control started recently to control OP and SP resistant ticks (as stated by farmers). But, upon the indiscriminate and incessant use for past few years, the problem of resistance against amitraz is emerging and would soon be widespread as the development of resistance is dependent on the frequency of resistant individuals in the population and the intensity of chemical selection pressure [13].

The current study reports only the detection of resistance status of *R. (B.) microplus* against amitraz and the mechanism involved needs to be established. The mode of action of amitraz is believed to be interference with nervous system function of the targeted pest species by binding to the octopamine receptors [14]. Further, technical grade amitraz was selected over commercial formulation for the bioassay as commercial products are prepared with many proprietary ingredients and it is difficult to assess the responses due to active ingredients [15]. The stock solutions were prepared by dissolving in 100% methanol as use of organic solvents facilitates the adsorption and penetration of active ingredients of the acaricide across the exoskeleton [6].

The results revealed development of resistance in *R. (B.) microplus* to amitraz from Punjab state. The data generated would be useful in development of effective control strategies against ticks. This will further prevent the development of resistance and at the same time will decrease environmental pollution, thus also causing reduction in the residual effect of acaricides in the animal products like milk and meat.

## Figures and Tables

**Table 1:** Dose-dependent response of amitraz against *R. *(*B.*)* microplus* collected from SBS Nagar, Punjab.

Concentration (ppm)	Number of ticks	Average weight ± SE (mg)	Number of dead ticks (mortality%)	Average egg mass ± SE (mg)	RI (average egg mass/average weight)	IO%
62.5	20	82.1 ± 3.48	0 (0)	32.3 ± 3.08	0.393	31.75
125	20	81.9 ± 3.68	8 (40)	27.6 ± 2.60	0.336	27.92
250	20	79.5 ± 3.77	8 (40)	25.1 ± 1.71	0.315	35.94
500	20	86.3 ± 3.62	14 (70)	27.6 ± 0.91	0.319	46.16
1000	20	85.5 ± 4.73	16 (80)	21.5 ± 0.67	0.251	52.73
Control	20	97.3 ± 6.22	0	42.0 ± 4.42	0.431	0.0
